# Suspected Aripiprazole-induced neutropenia in a geriatric patient: a case report

**DOI:** 10.1186/s12877-020-01514-x

**Published:** 2020-05-24

**Authors:** Tyler Torrico, Nakisa Kiai, Carlos Meza, Md Towhid Salam, Sara Abdijadid

**Affiliations:** 1 Department of Psychiatry, Kern Medical Center, 1700 Mount Vernon Avenue, Bakersfield, CA 93306 USA; 2 Department of Preventive Medicine, University of Southern California, 1975 Zonal Ave., Los Angeles, 90033 CA USA

**Keywords:** Adverse effects, Aripiprazole, Neutropenia, Geriatrics, Blood dyscrasia, Drug-induced, Elderly, Case report

## Abstract

**Background:**

Aripiprazole, a third-generation antipsychotic medication, has been used to treat a range of psychiatric disorders. According to the U.S. Food and Drug Administration’s prescribing information, the most common adverse reactions in adult patients in clinical trials (≥10%) were nausea, vomiting, constipation, headache, dizziness, akathisia, anxiety, and insomnia. While hematological adverse effects may occur with aripiprazole, there is very limited information in the published literature on such adverse outcomes.

**Case presentation:**

A 68-year-old Caucasian male with treatment resistant depression was hospitalized for suicidal ideation. The patient developed neutropenia after aripiprazole was introduced as an augmentation agent. The neutropenia was reversible with discontinuation of the medication.

**Conclusions:**

To our knowledge, we describe the first case report of suspected neutropenia-induced by aripiprazole use in a geriatric patient. While hematological adverse reactions are rare, we recommend adding CBC to the standard adverse systemic reaction monitoring of antipsychotic medications, particularly among the elderly.

## Background

Aripiprazole is a third-generation [also known as atypical] antipsychotic that acts as a partial agonist at dopamine receptors subtype 2 (D_2_), a partial agonist at serotonin 1A (5-HT_1A_) receptors and an antagonist at serotonin 2A (5-HT_2A_) receptors. Aripiprazole has been approved by the U.S. Food and Drug Administration (FDA) for treatment of schizophrenia, bipolar mania, Tourette’s disorder, irritability associated with autism spectrum disorder, and adjunct to antidepressants in treatment of major depressive disorder [1–3]. While neuropsychiatric (e.g., headache, akathisia, insomnia, somnolence), cardiometabolic (e.g., hyperglycemia, dyslipidemia, weight gain) and gastrointestinal (e.g., nausea, vomiting, salivary hypersecretion, etc.) are more common adverse reactions, hematological abnormalities associated with aripiprazole use are rare. Existing literature is limited, with documented neutropenia associated with aripiprazole use in children in three case-reports [4–6]; and some case reports in adults [6–12]. The FDA Adverse Event Reporting System (FAERS) Public Dashboard lists 10 suspected cases of aripiprazole-associated neutropenia in geriatric patients; but to our knowledge, there is no published literature describing aripiprazole-induced neutropenia in a geriatric patient. Informed consent was obtained from the patient about both participating in this study and for publication of this case report. The Kern Medical Institutional Review Board approved the study protocol.

## Case presentation

A 68-year-old Caucasian male was brought to the emergency department by local police due to suicidal ideation with plan and intent. The patient has a diagnosis of major depressive disorder with multiple involuntary psychiatric hospitalizations. The patient’s family history is unknown. He had been prescribed citalopram (20 mg daily) and bupropion (extended release 300 mg daily) upon discharge from a psychiatric hospitalization 1 year prior, but he had not been taking these medications prior to this admission due to self-reported lack of efficacy. The patient has multiple medical comorbidities including uncontrolled type 2 diabetes mellitus, hypertension, benign prostatic hyperplasia and chronic obstructive pulmonary disease. His medical conditions were being treated with his home medications, which include insulin glargine, insulin lispro, metformin (1000 mg daily), and lisinopril (20 mg daily).

On admission, his labs were unremarkable except for a mild normocytic anemia (hemoglobin: 12.6 × 10^3^, mean corpuscular volume: 88.6 μm^3^) and thrombocytopenia (108 × 10^3^) both of which he has had chronically for at least 2 years based on our records. His white blood cell count (WBC) was 4.5 × 10^3^, and his absolute neutrophil count (ANC) was 2.6 × 10^3^ on admission. On hospital day 1, he was started on his home medications and citalopram (20 mg daily) due to a positive response from a previous hospitalization. Bupropion was not used this hospitalization. Additionally, a Foley catheter was inserted due to urinary retention. The patient continued the same medication regimen and continued to have severe suicidal ideation for hospitalization days two through eight. On hospital day nine, the patient continued to endorse severe depressive symptoms and suicidal ideations and plan, necessitating more aggressive medication management to address these symptoms, which the patient agreed to. Levothyroxine 125 micrograms daily was added to his regimen. On day ten, further augmentation was started with aripiprazole 5 mg daily.

**Fig. 1:**
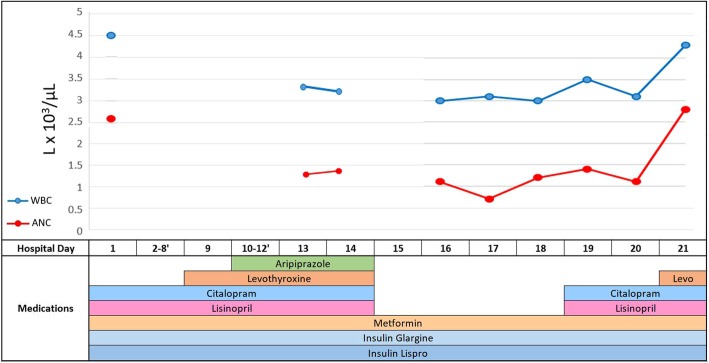
White blood cell (WBC) and absolute neutrophil count (ANC) by hospitalization day with medication regimen

The patient responded well to the augmented regimen based on daily assessments showing improved mood and decreased suicidality. However, on hospital day 13, hypotension prompted a lab work up due to concern for sepsis as the patient had an indwelling Foley catheter since admission. These labs (day 3 of aripiprazole use) were unremarkable except for WBC count of 3.3 × 10^3^ and ANC of 1.3 × 10^3^ (Fig. 1). Due to concerns of neutropenia, complete blood count (CBC) with differential was repeated the next day which showed no appreciable change. At this point, an internal medicine consult was sought and citalopram, aripiprazole, levothyroxine and lisinopril were held per consult recommendation (5 days of aripiprazole use total). Laboratory work up for other causes of blood dyscrasias included negative screenings for chronic infections [human immunodeficiency virus (HIV) and hepatitis], normal serum levels of albumin, vitamin B12 and folate. However, a positive antinuclear antibody (ANA) with nucleolar pattern and titer of 1:40 was found.

His WBC and ANC continued to demonstrate a downward trend for the following 2 days, to as low as 3.0 × 10^3^ for WBC and most concerning of all an ANC value of 0.7 × 10^3^ on day 17. Despite the abnormal CBC’s, the patient was asymptomatic for signs of infection throughout his hospitalization. On day 19, citalopram and lisinopril were restarted due to an uptrend in ANC. Levothyroxine was restarted once his ANC returned to baseline (2.8 × 10^3^) on day 21. By day 21, the patient was discharged as he was no longer endorsing suicidal thoughts, his neutropenia had resolved, and he was in remission from depression. No additional pro re nata (PRN) medications were administered this hospitalization.

## Discussion

This is the first descriptive case report of suspected aripiprazole-induced neutropenia in a geriatric patient. While a few cases of aripiprazole-induced neutropenia have been documented in the literature for younger age groups [4, 5, 7–10], the true incidence of this adverse reaction is not known as regular monitoring of CBC with aripiprazole use is not the current standard of care [13–15]. There is potential for concern, as neutropenia can be a life-threatening condition, especially in specific populations such as an elderly patient who may already be suffering from comorbid conditions and/or immunosuppression. Lab work to monitor for metabolic changes (fasting blood glucose and lipids) when starting atypical antipsychotics is the standard of care [15]. We recommend adding CBC to routine monitoring, as it is an inexpensive yet widely accessible test.

The diagnostic approach to neutropenia in an elderly patient is most efficiently investigated with a bone marrow biopsy, however, this may not always be clinically indicated if an etiology is able to be explained by patient history and/or basic laboratory panels [16]. A history of hereditary blood dyscrasias, and chronic infections should be inquired. Basic laboratory studies such as vitamin B12, folate, albumin, ANA, HIV screening, hepatitis panel should be obtained upon initial evaluation. If neutropenia is still unable to be explained, the first specific action should be discontinuation of all drugs not necessary for maintenance of vital functions [16]. If still unable to be explained, a bone marrow biopsy should be obtained [16]. In this case, the patient had no history of blood dyscrasias, chronic infections and basic laboratory panels were unrevealing. The patient did have a positive ANA with nucleolar pattern; therefore, it is possible that there was an autoimmune component to the patient’s neutropenia. However, the patient had no clinical correlation to the symptoms of the autoimmune diseases typically associated with a nuclear pattern (systemic lupus erythematosus, rheumatoid arthritis, Sjögren’s syndrome, scleroderma and systemic sclerosis, Raynaud phenomenon) [17–19]. Further, the interpretation of ANA patterns and titers is largely controversial when there is no clinical correlation, as healthy individuals (including the elderly) have been shown to have a positive ANA with various patterns and at various titers [17–19].

Instead, there are stronger reasons to associate the patient’s neutropenia with aripiprazole exposure as a temporal relationship was demonstrated. Application of the Naranjo adverse drug reaction probability scale results in score of + 4, categorizing the likelihood of causality as *possible* [20]. A consideration in determining causality was lacking from this study, re-challenging the suspected agent. However, in the few similar cases where patients were re-challenged with aripiprazole, they invariably developed neutropenia again [4–12, 21]. Additionally, given the risks associated with neutropenia (particularly in the context of patients who are hospitalized having higher risk of nosocomial infections), risks outweighed the benefits of re-challenging aripiprazole in this patient. Furthermore, the patient was clinically improving from his depression without aripiprazole at the time that re-challenge would have been warranted.

Although this patient was lost to follow up so no post-hospitalization lab work was able to be obtained, review of the literature of similar studies showed that both children and adults had their blood levels (WBC and ANC) uptrend within 3–4 days after discontinuing aripiprazole with baseline levels being achieved by 1 week [4–12, 21]. This trend is consistent with the patient described in this study as his blood levels (WBC and ANC) began to uptrend after the fourth day of discontinuing aripiprazole, with baseline levels achieved after 7 days. In the reported cases that included follow up laboratory studies after hospital discharge; at 2 weeks [5, 7], 4 weeks [5, 8], and 6 months [5]; WBC and ANC counts remained within normal limits with each post-hospitalization CBC. Given these previous findings, in patients who do not restart aripiprazole after an adverse neutropenic reaction occurs, follow up CBC’s does not appear to be necessary or revealing. Additionally, each case reviewed demonstrated a temporal relationship of neutropenia with exposure to aripiprazole [4–12].

It is highly unlikely that the patient developed the neutropenia from lisinopril or citalopram, as there is also no established association with neutropenia in either of these drugs [22, 23]. Although not required, a temporal relationship between an adverse reaction and exposure to the suspected agent is highly suspicious, and in this case a temporal relationship only exists with aripiprazole. Additionally, the patient did not have a prior adverse reaction to lisinopril or citalopram during previous hospitalizations and restarting the medications did not result in a return of neutropenia. Levothyroxine’s adverse effects are typically limited to symptoms of hyperthyroidism, and it is not known to be associated with neutropenia [24]. It is highly unlikely metformin or insulin induced the patient’s neutropenia as he has taken these medications for decades due to his chronic diabetes mellitus. Additionally, while the patient’s anemia and thrombocytopenia mildly fluctuated throughout this hospitalization, there was no significant change or trend away from baseline, suggesting that this was an isolated neutropenic dyscrasia.

**Fig. 2:**
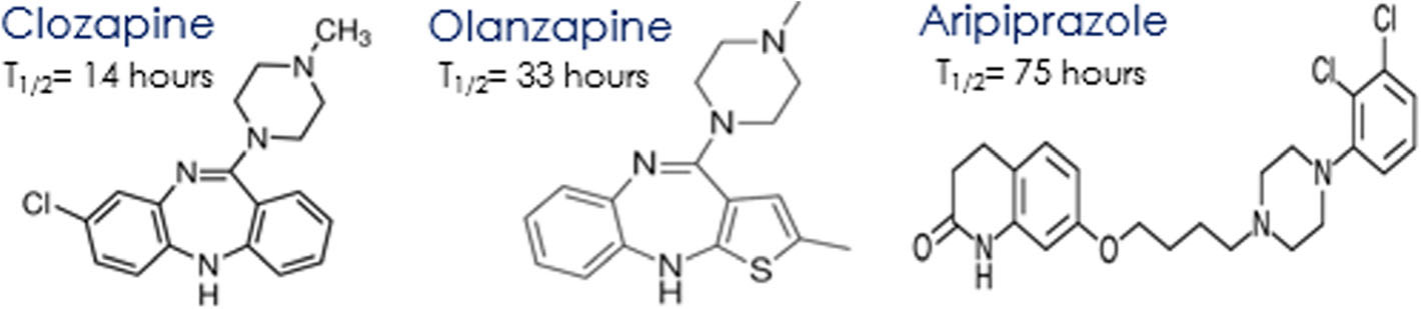
Contrast between the molecular structures and mean half-life’s (t_1/2_) of clozapine, olanzapine and aripiprazole

The exact mechanism for aripiprazole-associated neutropenia is unknown, and there are not enough reported cases to establish which populations may be at risk. However, clozapine induced agranulocytosis has been extensively studied and may partially explain the observed association in this case. Aripiprazole is like clozapine and olanzapine in that their metabolism involves the production of nitrenium ions [25]. In clozapine studies, these nitrenium ions were shown to covalently bind to neutrophil proteins and act as a hapten, initiating an immune-mediated destruction of affected neutrophils [26]. Additionally, the HLA-B38 phenotype appeared to be more often affected suggesting genetic elements are involved as well. Olanzapine has a similar molecular structure to clozapine (Fig. 2) and has a similar mechanism of action as they are both tricyclic atypical antipsychotics. However, neutropenia is less prevalent with olanzapine use compared to clozapine. This is likely due to olanzapine’s longer half-life resulting in less activation of the cytochrome P450 system and therefore less nitrenium ion formation [27]. Aripiprazole, although it is an atypical antipsychotic, it is not of the tricyclic class. Aripiprazole belongs to the phenylpiperazine class, and it has a markedly longer half-life than both clozapine and olanzapine [28]. This is likely a contributing reason for aripiprazole’s significantly improved safety regarding hematologic adverse reactions, when compared to other atypical antipsychotics [27].

In conclusion, we report occurrence of neutropenia in an elderly male who was prescribed aripiprazole to augment an antidepressant medication that was reversible with discontinuation of the medication. While hematological adverse reactions are rare, we recommend adding CBC to the standard adverse systemic reaction monitoring of antipsychotic medications, particularly among the elderly.

## Data Availability

The data displayed during the current study are not publicly available as they are part of protected health information of the patient described. But are available from the Kern Medical Center upon reasonable request and approval.

## Abbreviations

FDA: Food and Drug Administration
WBC: White blood cell
ANC: Absolute neutrophil count
CBC: Complete blood count
HIV: Human immunodeficiency virus
ANA: Antinuclear antibody
PRN: Pro re nata
